# Study on the Performance and Mechanism of Glass Fiber-Reinforced MgO-SiO_2_-H_2_O Cement

**DOI:** 10.3390/ma16206668

**Published:** 2023-10-12

**Authors:** Tingting Zhang, Jingbin Zhang, Yang Zhao, Hongmei Ai

**Affiliations:** Faculty of Infrastructure Engineering, Dalian University of Technology, Dalian 116024, China; tingtingzhang@dlut.edu.cn (T.Z.); zjb1001@mail.dlut.edu.cn (J.Z.); zhaoyang0429@mail.dlut.edu.cn (Y.Z.)

**Keywords:** glass fiber, magnesium silicate cement, mechanical properties, shrinkage performance, accelerate aging performance

## Abstract

The magnesium silicate hydrate system (MgO-SiO_2_-H_2_O) possesses issues such as susceptibility to cracking, brittleness, and poor volumetric stability, which hinder its development and practical use in engineering applications. This study aimed to enhance the properties of the MgO-SiO_2_-H_2_O system by incorporating glass fiber as a reinforcing material. The mechanical properties, shrinkage properties, and properties during accelerated aging were tested at different content levels of glass fiber. Additionally, the reaction mechanism and microscopic morphology were characterized using microscopic testing methods. The results revealed that the addition of glass fiber improved the mechanical properties of the MgO-SiO_2_-H_2_O system; meanwhile, with an increase in fiber content, the mechanical properties showed an initial increase followed by a decreasing trend. With a glass fiber content of 0.6%, the system exhibited a flexural strength of 7.9 MPa at 28 d, a compressive strength of 42.5 MPa at 28 d, and a 27.2% increase in splitting tensile strength compared to the control group. At a fiber content of 0.9%, the flexural toughness steadily increased, reaching a maximum value of 2.238 N·m, which is 5.41 times greater than that of the control group. Moreover, the incorporation of glass fiber effectively inhibited the shrinkage of the MgO-SiO_2_-H_2_O system. Accelerated aging experiments confirmed that the glass fiber in the MgO-SiO_2_-H_2_O system did not undergo significant deterioration or corrosion, thereby maintaining long-term stability. These findings have important theoretical and practical significance for the application and development of the MgO-SiO_2_-H_2_O system.

## 1. Introduction

The accelerated process of industrialization in the construction industry has led to a significant rise in the demand for cement. Global annual cement production has surpassed 3 billion tons, resulting in CO_2_ emissions accounting for approximately 8% of per capita CO_2_ emissions annually [1]. China currently holds the dubious distinction of being the world’s largest emitter of CO_2_; by the end of 2022, China’s cement blending capacity reached 2.118 billion tons. Traditional cement production not only emits a substantial amount of CO_2_ but also consumes nonrenewable resources and valuable electrical energy. The production process of magnesium cementitious material offers a simple and relatively low overall CO_2_ emission solution, making it one of the key areas of research for green and low-carbon cement. The MgO-SiO_2_-H_2_O system is one type of magnesium cementitious material and is characterized by its lightweight, low alkalinity, large specific surface area, and resistance to corrosion. These properties make it highly suited for various applications, including the solidification of nuclear waste [2,3], the adsorption of heavy metal ions [4,5,6], and its use as a thermal insulation material [7,8].

In 1953, W.F. Cole [9] proposed the concept of magnesium silicate hydrate (MgO-SiO_2_-H_2_O) after discovering a white precipitate in severely damaged concrete breakwater and identified the molecular formula of the substance as 4MgO·SiO_2_∙8.5H_2_O. Subsequently, Wunder et al. [10] established a phase diagram for the ternary cementitious, system of MgO-SiO_2_-H_2_O and analyzed the different reaction product phases within it. Both domestic and international scholars have conducted related studies on the preparation, synthesis [11,12,13,14,15,16,17,18], microstructure [16,19,20,21,22], and reaction mechanism [23,24,25] of the MgO-SiO_2_-H_2_O system. The magnesium silicate hydrate binder gels (M-S-H gels) have been investigated by C. Roosz et al. [20] and M. Tonelli et al. [22] through techniques such as solid-state nuclear magnetic resonance (NMR) and X-ray diffraction (XRD) tests. The results have shown that the M-S-H gel has a layered silicate structure. The Mg/Si molar ratio for the preparation of M-S-H gels was found to range from 0.67 to 1, according to studies conducted by Brew et al. [16]. Moreover, Zhang et al. [26] discovered that the addition of 1% sodium hexametaphosphate (Na-HMP) significantly reduced the water requirement of the MgO-SiO_2_-H_2_O system and greatly increased the compressive strength, exceeding 70 MPa. Li et al. investigated the effects of raw material properties, curing temperature, and water–cement ratio (w/c) on the hydration of the MgO-SiO_2_-H_2_O system [27,28].

Currently, there is extensive research on fiber-reinforced cement and concrete composite materials. Studies have demonstrated that the incorporation of fibers into cement mortar can enhance its mechanical strength and durability while addressing issues such as cracking and brittleness. Various types of fiber-reinforced cement mortar composites are now widely utilized. Researchers have explored the impact of steel fiber dimensions [29], content [30], and distribution patterns [31,32] on cement mortar. The findings indicate that the addition of steel fibers can improve the mechanical strength and workability of cement and enhance the toughness of ultra-high-performance concrete [33]. Polyethylene fiber is also commonly employed in fiber-reinforced cement-based composites. Bi Yutao et al. investigated the influence of ultra-high molecular weight polyethylene fibers on the carbonation resistance and freeze–thaw resistance of cement-based composites [34]. Also, study [35] highlighted that blending steel fibers and polyethylene fibers in appropriate proportions can enhance the compressive strength of cement-based materials and proposed a constitutive model. With the increasing demand for ecological construction, natural fibers have garnered significant attention in the cement industry. Incorporating basalt fibers into cold recycled asphalt mixtures can improve their resistance to cracking and hinder crack propagation [36]. Furthermore, basalt fibers also possess long-term strengthening effects on ordinary Portland cement (OPC) and high-belite sulfoaluminate cement (HB-CSA) [37]. Additionally, various fibers, including basalt, sugarcane bagasse, and cotton, can be widely utilized in cement and geopolymer concrete [38].

Glass fiber, as an inorganic non-metallic material, is commonly added to concrete or cement to enhance its compressive strength, resistance to splitting forces, and overall structural performance [39]. A study conducted by Ribeiro et al. [40] examined the impact of glass fiber waste content, length, and the addition of fly ash to ordinary Portland cement (OPC). The research findings revealed that the inclusion of glass fiber reinforces the mechanical properties of OPC and reduces crack propagation within the cementitious material. Chen et al. [40] conducted a similar investigation on glass fiber cement and found that it can improve the resistance to tensile strength and low toughness characteristics of OPC, thereby enhancing its overall performance. Introducing glass fiber into magnesium phosphate cement systems can notably improve the mechanical properties of the cement. However, it is worth mentioning that increasing the fiber content and length can result in a decline in mechanical performance [41]. In conclusion, while glass fiber can enhance the mechanical properties of cement and concrete, as well as address concerns such as volume stability and cracking, it is important to note that the low-alkalinity environment in the OPC pore solution can lead to corrosion of the glass fibers, negatively impacting its structure and performance [40].

The MgO-SiO_2_-H_2_O system exhibits a relatively low pH environment (pH around 10 at 28 d), so many fibers have good chemical compatibility with the M-S-H matrix, and fiber reinforcement is a simple and effective method to improve the ductility and toughness of the MgO-SiO_2_-H_2_O system. Marmol, G. and Savatano, H. [42] demonstrated through four-point bending tests that the MgO-SiO_2_-H_2_O system and cellulose fibers have good compatibility. Zhang et al. [43,44] incorporated 1–5 wt% of alkali magnesium sulfate whiskers into the MgO-SiO_2_-H_2_O system, resulting in improvements in both compressive strength and flexural strength. Sonat [45] incorporated 2% of polyvinyl alcohol fibers (PVA) into the MgO-SiO_2_-H_2_O system to produce a new strain of hardening magnesium–silicate–hydrate composites (SHMSHC), which has the potential to broaden the application of the MgO-SiO_2_-H_2_O system. Zhang et al. [46] utilized high-strength and tough feather fibers to reinforce M-S-H gels, for which the MgO-SiO_2_-H_2_O system has the potential to produce boards with low density, high strength, and low thermal conductivity. Li et al. [47] investigated the aging resistance of lignocellulosic fiber-reinforced magnesium silicate hydrate cement and found that, with increased aging time, the flexural strength of the cement with lignocellulosic fibers surpassed that of ordinary cement. Wang et al. [48] conducted research on the enhancement and modification of the MgO-SiO_2_-H_2_O system using bamboo pulp fibers, examining the influence of bamboo pulp fiber content on the flexural strength and fracture toughness of MgO-SiO_2_-H_2_O cement.

The studies referenced indicate that the inclusion of fibers in the MgO-SiO_2_-H_2_O system enhances its mechanical properties and reduces system shrinkage while minimizing the erosion and degradation of the fibers in the cement matrix, and the long-term reinforcement effect of the fibers is more stable, resulting in a significant improvement in durability. However, there is currently a lack of research specifically focusing on the use of glass fiber reinforcement in MgO-SiO_2_-H_2_O cement. This study investigated the preparation of glass fiber-reinforced magnesium silicate hydrate cement and explored the impact of different glass fiber contents on the mechanical properties, shrinkage properties, and properties under accelerated aging conditions. In conjunction with microtesting methods, the study aimed to characterize the reaction mechanism and microscopic morphology to optimize the performance and application of the MgO-SiO_2_-H_2_O system.

## 2. Materials and Methods

### 2.1. Materials and Sample Preparation

The raw materials utilized in the preparation of glass fiber-reinforced magnesium silicate hydrate cement-based composite materials mainly consist of commercially available conventional materials, such as magnesia (MgO), silica fume (SF), sodium hexametaphosphate (NaHMP), quartz sand, and glass fiber. The chemical composition and physical properties of the raw materials are shown in Table 1. The MgO employed is light-burned active magnesia obtained from Xinhua Metal Materials Company in Xingtai City, Hebei Province of China, with an active MgO content of 86.7%, as tested by the hydration method, and an activity result of 22.98 s, as examined using the citric acid method, indicating its high activity. The silica fume used is Elkem 940 U microsilica powder, manufactured by Elkem in Shanghai, China, with a D50 particle size of 234 nm. The quartz sand was sourced from Xinlian Quartz Sand Factory in Zhuanghe, Liaoning Province of China, and features a D50 particle size of 117.63 μm. Sodium hexametaphosphate constitutes an analytical pure chemical reagent procured from China National Pharmaceutical Chemistry Reagent Corp. The glass fiber utilized is alkali-resistant glass fiber, produced by Taishan Glass Fiber Company in Tai’an City, Shandong Province of China.

The mixing ratio for the glass fiber-reinforced magnesium silicate hydrate cement materials prepared in this study is provided in Table 2. The preparation process for glass fiber-reinforced MgO-SiO_2_-H_2_O cement mortar is as follows: Firstly, the raw materials, including MgO, SF, and quartz sand, were accurately weighed and added to a mixing pot. The measured raw materials were then mixed at a low speed for 2–3 min until a uniform mixture was obtained. Next, the pre-dissolved solution of sodium hexametaphosphate was poured into the mixer and stirred at a low speed for 3 min until a flowable slurry formed. After thorough stirring, the glass fibers were slowly added to the mixing pot while continuously stirring at a fast speed for 3 min. Finally, the well-mixed cement slurry was poured into molds and compacted through vibration. After curing at room temperature for 24 h, the molds were removed, and the labeled specimens were transferred to a standard curing environment (with a temperature of 20 ± 2 °C and a relative humidity of 95%) for further curing up to 28 days.

### 2.2. Methodology

#### 2.2.1. Measurement of Mechanical Performance

The bonding between glass fibers and the MgO-SiO_2_-H_2_O cement matrix, as well as the interfacial bonding forces between them, contribute to the improvement in the mechanical properties of cement-based composite materials. In order to analyze the impact of glass fibers on the mechanical properties of cement-based materials, various tests were conducted, including flexural strength, compressive strength, flexural toughness, and splitting tensile strength of the cement-based composite materials.

The flexural strength was tested in accordance with the “Strength Testing Method for Cement Mortar (ISO Method)” (GB/T17671-1999) [49] using specimens measuring 40 mm × 40 m × 160 mm. The testing procedure utilized the WHY-300/10 microcomputer-controlled pressure testing machine from Shanghai Hualong Company, Shanghai, China, with a loading rate of 50 N/s until fracture occurred. Each group prepared three specimens for flexural strength testing, and the average value was taken as the result of compressive strength testing.

For the compressive strength test, the specimens that had undergone the flexural strength testing were placed into a compression fixture. A loading rate of 2400 N/s was applied to an area measuring 40 mm × 40 mm until the specimens were destroyed. Six specimens were used for each group, and the average value was taken as the result of the compressive strength.

The splitting tensile strength test was conducted following the standard in the JTG3420-2020 [50] “Test Code for Cement and Cement Concrete in Highway Engineering”. Cube-shaped specimens with dimensions of 70.7 mm × 70.7 mm × 70.7 mm were used to measure the splitting tensile strength. The splitting tensile test was performed using the WHY-300/10 microcomputer-controlled pressure testing machine at a loading rate of 0.1 kN/s. Three specimens were used for each group, and the average value was taken as the test result.

The determination and calculation of flexural toughness were conducted using a universal testing machine, specifically Model E45. The specimens used for the experiment had dimensions of 40 × 40 × 160 mm, and the three-point bending test method was employed. Linear variable differential transducers (LVDT) were symmetrically placed on both sides of the specimens to measure the deformation at the center as deflection at the mid-span. The loading rate was set at 0.5 mm/min, and the span of the specimens was 120 mm. The flexural toughness was calculated according to the JCI-SF4 standard [51]. The load-deflection curve within a specific deflection range was integrated using Equation (1) to obtain the flexural toughness.

(1) $$T_{b}=\int_{0}^{\delta }P\left(\sigma \right)d\sigma$$

*T_b_* is the flexural toughness, N·m.

*δ* is typically calculated as *δ* = 2.4 mm (L/50, L = 120 mm). Notably, in some cases during the experiment, the specimens failed before reaching a *δ* of 2.4 mm, resulting in the inability to measure the flexural load. In these cases, the value of *δ* is taken as the deflection at which *P*(*σ*) = 0.

*P*(*σ*) represents the flexural load.

#### 2.2.2. Shrinkage Testing

The MgO-SiO_2_-H_2_O system is prone to volumetric deformation and cracking. In cementitious materials, fibers can play a bridging role in suppressing shrinkage deformation. In order to investigate the improvement in shrinkage in the MgO-SiO_2_-H_2_O system caused by the addition of glass fibers, the shrinkage of the glass fiber-reinforced MgO-SiO_2_-H_2_O cement was tested under conditions of drying and sealing.

**Figure 1:**
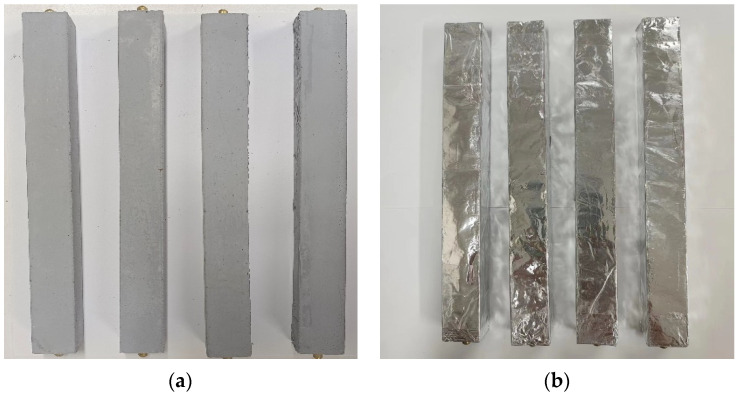
The shrinkage testing samples: (**a**) the drying shrinkage sample and (**b**) the shrinkage sample under sealed conditions.

The dry shrinkage measurement was conducted following the standardized method JC/T603-2004 [52] “Test Method for Shrinkage of Cement Mortars”. The shrinkage specimens were sized at 25 mm × 25 mm × 280 mm, and after the specimens were molded, they were placed in a standard curing room for 24 h before being demolded. Subsequently, the specimens were immersed in water for 48 h, and the surface moisture and any impurities on both sides were wiped away using copper tops. The initial length value was measured using a length comparator; after measuring, the specimens were subjected to the drying condition (20 °C, RH = 50%). The length of the specimens was measured at various ages, and three specimens were tested for each group, with the average value taken as the test result. The dry shrinkage specimens are illustrated in Figure 1a.

The shrinkage measurement under sealed conditions followed the methods described in references [53,54]. The dimensions of the test specimens were uniformly set at 25 mm × 25 mm × 280 mm. After being cured under standard conditions for 24 h, the specimens were demolded and then immersed in water for 48 h. After removal from the water, the surfaces were carefully wiped to eliminate any moisture and impurities from the copper nails on both sides. The initial length was measured using a length comparator. Then, the specimens were sealed by wrapping them with double-layered aluminum foil and paraffin, as shown in Figure 1b, to ensure that the internal moisture of the mortar did not evaporate or dissipate into the surroundings.

The shrinkage of the specimen is calculated according to the following formula:

(2) $$\varepsilon_{i}=\frac{L_{i}-L_{0}}{L}\times 100\%$$

(3) $$M_{i}=\frac{m_{0}-m_{i}}{m_{0}}\times 100\%$$

In the given equation, ε_i_ represents the shrinkage rate of specimen i on the i-th day, calculated from the initial length of the test, expressed as a percentage (%). L_0_ refers to the initial length of the specimen, measured in millimeters (mm), after being removed from water and dried to eliminate surface moisture. L_i_ represents the length measurement of the specimen obtained on the i-th day of aging, in millimeters (mm). L denotes the effective length of the specimen, calculated as 250 mm.

M_i_ indicates the mass loss rate of the specimen on the i-th day, expressed as a percentage (%). m_0_ represents the original mass of the specimen, measured in grams (g), and mi represents the mass of the specimen on the i-th day, also measured in grams (g).

#### 2.2.3. Test of Fluidity

The incorporation of fibers disrupts the original particle motion equilibrium in cementitious materials, leading to an increase in the yield stress required for particle movement and consequently resulting in a decrease in the flowability of the paste. Therefore, it is crucial to test the flowability.

The fluidity of cement is determined using the method specified in GB/T 2419-2005 [55] “Method for Determination of Flowability of Cement Mortar”. The uniformly mixed mixture is divided into two portions and placed into a truncated cone mold. The first portion is filled to 2/3 of the height of the mold and compacted using a pestle, and then the remaining mixture is added to the mold and compacted. Excess mixture is gently scraped off with a scraper. The mold is then lifted lightly and subjected to 25 vibrations on a vibration table. The diameter of the mixture in two vertical directions is measured, and the average value is taken as the fluidity extension of the mixture.

#### 2.2.4. Accelerated Aging Tests

Compared with OPC, MgO-SiO_2_-H_2_O system has the advantage of being a long-term matrix of glass fiber due to its low alkalinity in the hydration process. In order to investigate the long-term durability of glass fibers in MgO-SiO_2_-H_2_O cement and the potential for enhancing toughness, accelerated aging tests were conducted. The accelerated aging tests are according to the SIC (strand in cement) method proposed by Litherland et al., which assumes that the decrease in strength is a result of chemical attack by the cement paste on the glass fiber, and through investigating the strength loss of specimens under high-temperature (50 °C or 80 °C) water-curing conditions, the strength loss of specimens exposed to a 20 °C environment for a prolonged duration can be predicted. The SIC method was utilized to investigate the flexural and compressive strength of glass fiber-reinforced magnesium silicate hydrate cement specimens under hydrothermal curing conditions at 20 °C, 50 °C, and 80 °C to assess changes in their performance under accelerated aging conditions.

#### 2.2.5. Microstructural Characterization

1. X-ray diffraction spectroscopy (XRD) was primarily utilized to investigate the hydration process of MgO-SiO_2_-H_2_O cement during accelerated aging and characterize its hydration products. It was used for qualitative and quantitative analysis of crystalline phases in the samples. The experiment employed a Bruker AXSD8 advanced type D8 Discover X-ray diffractometer from Mannheim, Germany. The instrument’s performance parameters were as follows: It utilizes a Cu target and a ceramic X-ray tube, along with a LynxEye detector and a vertical goniometer. The tube voltage was set at 40 kV, with a working current of 40 mA. The scanning speed was 0.5°2θ/min, and the 2θ angle scan range spanned from 5° to 80°.

2. X-ray fluorescence spectroscopy (XRF) was primarily utilized for the quantitative analysis of elements or oxides in raw materials. It was employed to investigate the hydration process of MgO-SiO_2_-H_2_O cement and characterize its hydration products during accelerated aging. In this experiment, the XRF-1800 X-ray spectroscopy instrument, manufactured by Shimadzu Corporation in Kyoto, Japan, was employed. The instrument was equipped with the following key performance parameters: a 4 kW thin window, Rh target, analysis diameter of 500 μm, scanning speed of 300° min^−1^, and a maximum current of 150 mA.

3. Thermogravimetric analysis (TGA) was used to characterize the hydration process and hydration products of MgO-SiO_2_-H_2_O cement during accelerated aging tests. The analysis was performed using a TGA-DSC 1 simultaneous thermal analyzer manufactured by METTLER-TOLEDO (Columbus, OH, USA). The main parameters involved in the analysis were a balance sensitivity of 0.01 μm, alumina crucibles, a testing temperature range of 50 to 800 °C, a heating rate of 10 °C/min, and an N_2_ atmosphere.

4. A mercury intrusion porosimeter (MIP) was adopted to test the pore structure of the samples. In order to investigate the influence of fiber content on the pore structure of cement-based materials and indicate the relationship between pore structure and mechanical strength, the pore structure of the specimens was characterized. The AutoPore IV9500 automatic analyzer, manufactured by Micromeritics Instrument Corporation in Norcross, GA, USA, was used to measure the pore size distribution and porosity of the specimens. The working parameters of the instrument included a pressure range of 0.02 psi to 50 psi and a measurable pore size range of 0.005 to 1100 μm.

5. A scanning electron microscope (SEM) was employed to analyze the microscopic morphology of the samples, which effectively characterized the dispersion degree, binding state, and microstructure of glass fibers in the matrix. The equipment used was the Nova Nano SEM-50, manufactured by FEI Company in Hillsboro, OR, USA. The specific working parameters of the instrument were as follows: an operating voltage of 230 V, an operating current of 8 A, a scanning frequency of 50/60 Hz, and a resolution of 1.5 nm and 1.0 nm in high and low vacuum modes, respectively.

## 3. Results

### 3.1. The Mechanical Properties

#### 3.1.1. Flexural Strength

**Figure 2:**
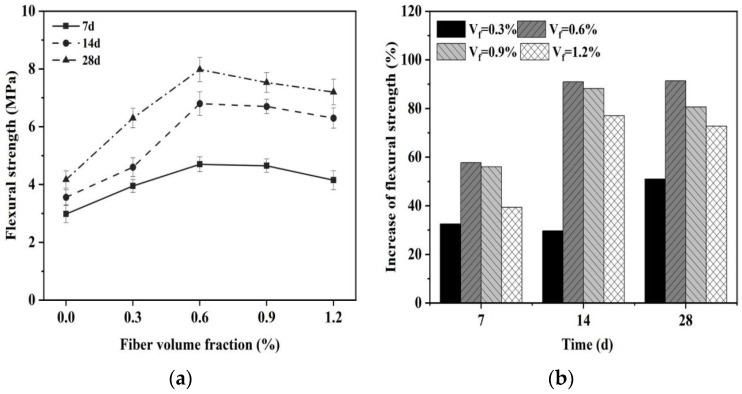
Effect of fiber content on flexural strength of magnesium silicate cement mortar. (**a**) Flexural strength and (**b**) increase in flexural strength.

Figure 2 illustrates the impact of glass fiber content on the flexural strength of the MgO-SiO_2_-H_2_O system. Figure 2a illustrates that the flexural strength of MgO-SiO_2_-H_2_O cement increases as the curing time increases, indicating the hydration process of cement-based composite materials. Figure 2b shows that the early-age flexural strength (7 d) of cement-based composite materials has a relatively slow growth rate, whereas the strength growth rate in the later stage (14–28 d) improves. This phenomenon is associated with the hydration process of MgO-SiO_2_-H_2_O. During the early stage of cement hydration, the main reaction is the formation of Mg(OH)_2_ from MgO. Although some M-S-H gel is also formed, its content is relatively low, thus making a minimal contribution to the development of strength in cementitious materials. As hydration proceeds, the amount of M-S-H gel increases, becoming the primary contributor to the strength of MgO-SiO_2_-H_2_O. Hence, the rate of flexural strength increase in the later stage is higher.

The flexural strength of the cement increases initially and then decreases with the increase in glass fiber content. In particular, the flexural strength is the highest for the sample with a glass fiber content of 0.6%, measuring 4.3 MPa and 7.9 MPa at the ages of 7 d and 28 d, respectively, which represents an increase of 46% and 91.4% compared to the control group. When the glass fiber content is relatively low (<0.6%), the fibers can be evenly distributed in the cement matrix, resulting in a toughening effect through the formation of a network structure [56]. The addition of glass fibers enhances the bonding interaction between the fibers and the MgO-SiO_2_-H_2_O cement matrix, thereby increasing the adhesive force at the fiber–matrix interface. This makes it difficult for the fibers to be pulled out of the cement matrix, resulting in an increase in the flexural strength of the cement. Within an appropriate range, as the fiber content increases, the bonding interaction between the fiber and the cement matrix strengthens, leading to an increase in flexural strength [57]. However, further increasing the fiber content (>0.6%) does not lead to a significant improvement in flexural strength. With the increase in fiber content, it becomes increasingly difficult for glass fibers to disperse in the cement matrix, resulting in agglomeration. The fibers become intertwined, thus reducing their bridging effect and leading to a decrease in flexural strength. Moreover, the increase in fiber dosage can result in a decrease in the flowability of the cementitious material (as observed in Section 3.1.2), leading to an increase in structural defects, which also contributes to a decrease in mechanical performance [29,31].

#### 3.1.2. Flexure Toughness

**Figure 3:**
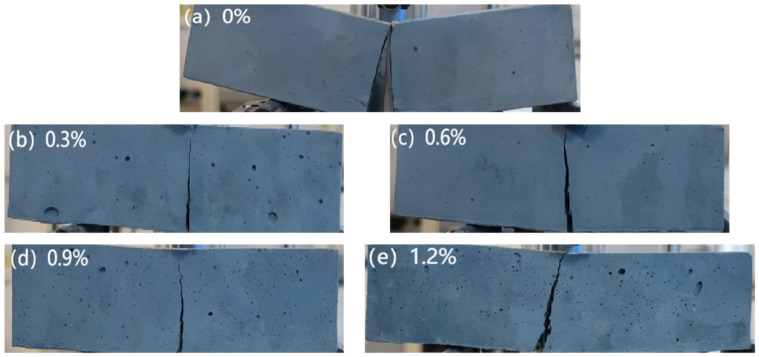
Cracking of MgO-SiO_2_-H_2_O mortar specimens with different fiber contents after 28 d flexural test.

**Figure 4:**
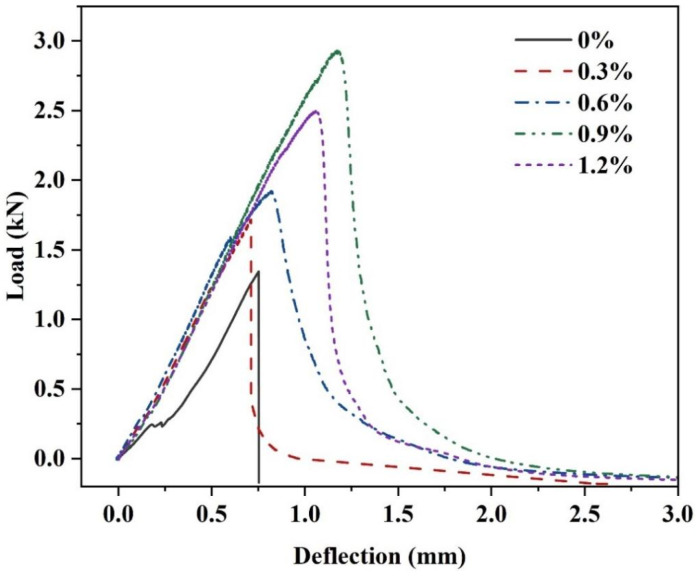
Load–deflection curve of MgO-SiO_2_-H_2_O mortar with different fiber contents at 28 d.

The incorporation of glass fibers can affect the post-failure morphology of the MgO-SiO_2_-H_2_O cement. As shown in Figure 3, the specimens without fibers exhibit clear brittle failure, with the specimens splitting into two halves upon reaching failure. Conversely, the specimens with fibers exhibit crack formation during failure but do not split into two halves, indicating that the brittleness of failure has improved. The load–deflection curves obtained from the three-point bending tests of fiber reinforced with varying fiber contents (Figure 4) reveal that the control group without fibers experiences a rapid decrease in load and fast crack propagation leading to failure upon reaching the maximum deflection value, indicating brittle failure. However, there is a slight increase in both peak load and deflection after the addition of fibers, with the load–deflection curve initially displaying a linear relationship. And after reaching the peak load, the load gradually decreases, suggesting that specimens with added glass fibers exhibit ductile fractures.

Table 3 summarizes the peak load, deflection values, and flexural toughness of magnesium silicate hydrate cement with varying fiber contents. The results indicate that the inclusion of glass fibers enhances the flexural toughness of the MgO-SiO_2_-H_2_O system. As the glass fiber content increases from 0% to 0.9%, there is a gradual increase in the peak deflection of the specimens. At a fiber content of 0.9%, the peak deflection reaches 1.098 mm, representing a significant 46.4% improvement compared to the control group. Additionally, the flexural toughness steadily increases until reaching a maximum value of 2.238 N·m, which is 5.41 times greater than that of the control group. The incorporation of glass fibers notably enhances the flexural toughness of the specimens by effectively suppressing cracks in the cementitious material and allowing the fibers to bear a portion of the applied stress. Consequently, the flexural toughness of the cementitious material is greatly improved. However, as the glass fiber content exceeds 1.2%, both the peak load and deflection begin to decrease, indicating a decrease in the toughening effect of glass fibers on the MgO-SiO_2_-H_2_O cement at high fiber contents. This decrease can be attributed to an excessively high fiber content, which adversely affects the overall integrity and crack resistance of the cement-based composite material, resulting in a reduction in toughness.

#### 3.1.3. Compressive Strength

**Figure 5:**
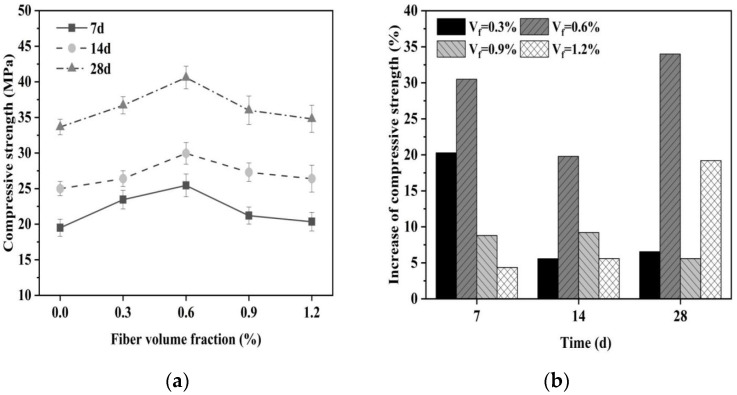
Effect of fiber content on compressive strength of magnesium silicate cement mortar. (**a**) Compressive strength and (**b**) increase in compressive strength.

The addition of glass fibers to the MgO-SiO_2_-H_2_O system has been found to improve the compressive strength. Figure 5 illustrates that the compressive strength of the specimens initially increases and then decreases with an increase in fiber content. Notably, when the fiber content is 0.6%, the specimens exhibit the highest compressive strength, reaching 42.5 MPa at 28 d, which represents a 34.3% increase compared to the control group. For fiber contents below 0.6%, the compressive strength of the specimens increases significantly due to the increase in the randomly distributed fibers inside the specimens, which can provide additional load-bearing capacity and increase the lateral constraint imposed on the material during compression, ultimately improving the compressive strength. An optimal amount of fibers can efficiently interlock with the hydration products during the hydration process, resulting in the formation of a network-like structure. This interlocking enables the effective transfer and dispersion of loads, consequently enhancing the compressive strength of the cementitious material.

However, the compressive strength does not continue to increase indefinitely with increasing fiber content. Instead, when the fiber content exceeds 0.6% and reaches 1.2%, the compressive strength at all testing ages experiences a significant decline. This decrease can be explained by a critical threshold existing between the fibers and the cement matrix, and exceeding this critical value will result in uneven dispersion of the fibers in the cement matrix, which can lead to a decrease in compressive strength. At the same time, an excessive inclusion of fiber can lead to the formation of stress concentration areas due to the intertwining of fibers. As a result, weakened regions are created under compression, leading to an uneven distribution of stress and subsequently impacting the overall strength of the material [58,59].

#### 3.1.4. Splitting Tensile Strength

**Figure 6:**
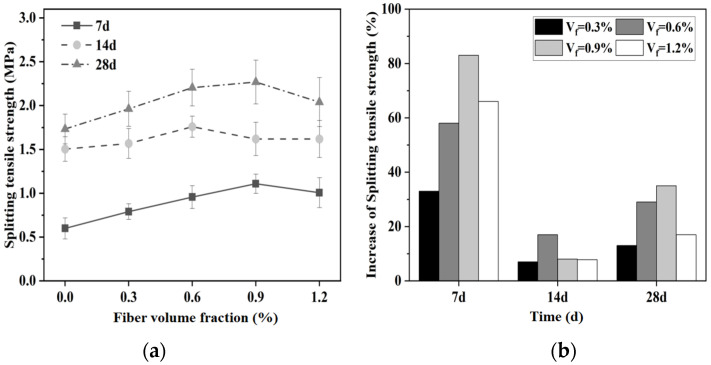
Effect of fiber content on splitting tensile strength of magnesium silicate cement mortar: (**a**) splitting tensile strength and (**b**) increase in strength.

The addition of glass fibers significantly improves the tensile performance of magnesium silicate hydrate cement-based composite materials. This improvement is evident in Figure 6, which shows that the splitting tensile strength of the MgO-SiO_2_-H_2_O system increases when glass fibers are added compared to the control group. After a curing period of 28 d, the splitting tensile strength of specimens with a fiber content of 0.3%, 0.6%, 0.9%, and 1.2% respectively increased by 13.3%, 27.2%, 30.6%, and 17.9% compared to the baseline group.

Upon the addition of glass fibers to the cement matrix, the fibers become surrounded by the cement as it hardens. This interaction generates frictional forces at the interface between the glass fibers and the cement matrix. Consequently, when the specimen is subjected to tensile stress, the weaker regions are the first to experience failure. At this stage, the glass fibers begin to bear the tensile load, effectively impeding the further propagation of cracks and enhancing the resistance of the specimen to splitting under tensile forces [45]. Furthermore, the presence of glass fibers also restricts the development of cracks in the cementitious material. The addition of fibers effectively bridges tensile cracks, transfers stress on both sides of the cracks, and delays crack propagation, resulting in improved splitting tensile strength. At lower fiber contents, the bridging effect of fibers is not effectively utilized, resulting in limited enhancement in splitting tensile strength. However, as the fiber content increases, the bridging effect becomes more prominent, resulting in a notable increase in splitting tensile strength. Furthermore, the splitting tensile strength of fiber-reinforced MgO-SiO_2_-H_2_O cement specimens increases with the curing age. This is because the bonding strength between the cement matrix and fibers gradually increases, thereby continuously improving the ability of the specimens to resist splitting. The bridging effect of fibers in the cement and the strong interfacial bonding between fibers and the matrix prevent the specimens from undergoing brittle failure.

### 3.2. Workability and Microstructural Characteristics

#### 3.2.1. Workability

**Figure 7:**
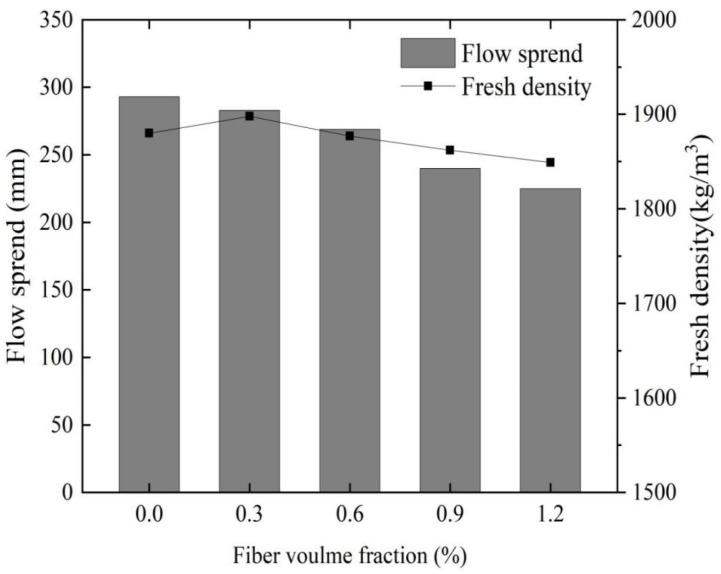
Effect of glass fiber content on fluidity and apparent density of magnesium silicate cement.

The study reveals that an excessive fiber content significantly reduces the fluidity of fresh cement, leading to poor dispersion of fibers and potentially leading to a deteriorated fiber reinforcement effect. Accordingly, it is imperative to examine the fluidity of fiber-reinforced cement in its fresh state [60]. Figure 7 illustrates the impact of glass fiber content on the workability and apparent density of MgO-SiO_2_-H_2_O cement. Notably, compared to the glass fiber-reinforced cement, magnesium silicate hydrate cement devoid of glass fibers exhibits superior fluidity. As the fiber content increases, the flow spread diameter of the mortar gradually diminishes from 290 mm. At a glass fiber content of 1.2%, the fluidity deteriorates to its lowest level with a minimum flow spread diameter of 228 mm, representing a 21.3% reduction compared to the reference group. Furthermore, the decrease in spread diameter becomes more pronounced with an increase in fiber content. These findings indicate that the incorporation of glass fibers adversely affects the fluidity of MgO-SiO_2_-H_2_O cement. The primary cause of this reduced fluidity can likely be attributed to the random and uniform distribution of glass fibers in the MgO-SiO_2_-H_2_O system, which forms a network structure acting as a “skeleton” within the cement matrix. Consequently, the movement of the cement slurry within the network structure is restricted, resulting in heightened friction and shear forces between the components of the mixture. These factors impede the free flow of the mixture and, consequently, negatively impact the fluidity of the mortar [61].

Additionally, under the premise of a constant water-to-cement ratio, the content of free water within the mixture is also constant. When fibers are incorporated into the mortar, a thin water film forms on the surface of the fibers during the mixing process. As the cement undergoes the hardening process, the relatively coarse surface of the fibers retains a certain amount of free water and gel products, which leads to a decrease in the effective w/c ratio in the early hydration process of cement-based composite material. Additionally, the roughness of the fibers increases the yield stress of the mortar particles and impedes the movement of fibers, thereby reducing the workability and flowability of the cement-based composite material. However, in contrast to natural fibers, glass fibers have a lower water absorption rate. Although some free water may be adsorbed on the surface, this can affect the flowability of freshly mixed cement by reducing the effective w/c ratio. Nonetheless, as cement continues to hydrate, the internal humidity decreases, and in the meantime, the absorbed free water by the fibers is released to inhibit the decline in internal humidity and has no impact on the strength of the hardened specimens. The primary factor responsible for the strength enhancement remains the incorporation of fibers [62].

Furthermore, the addition of glass fibers to magnesium silicate hydrate cement mortar slightly decreases the apparent density, shown in Figure 7, with the decreasing trend becoming more pronounced as the fiber content increases. The lower density of glass fibers compared to magnesium silicate hydrate cement, along with incomplete fiber dispersion during mixing, contributes to the development of internal voids and increased porosity of the test specimens.

#### 3.2.2. Pore Structure Characteristics

**Figure 8:**
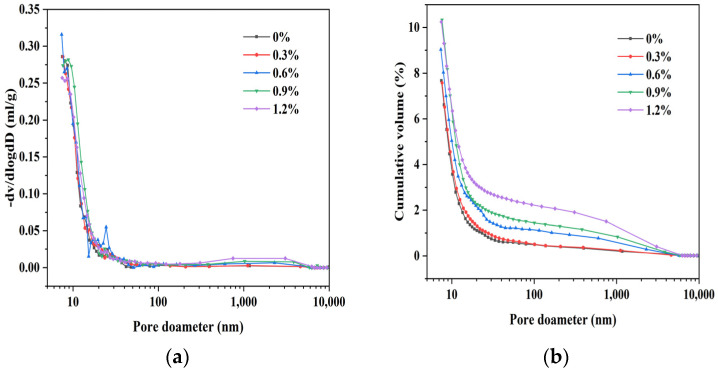
Pore size distribution of M-S-H with 0–1.2% glass fiber content. (**a**) Pore size distribution and (**b**) cumulative pore volume.

**Figure 9:**
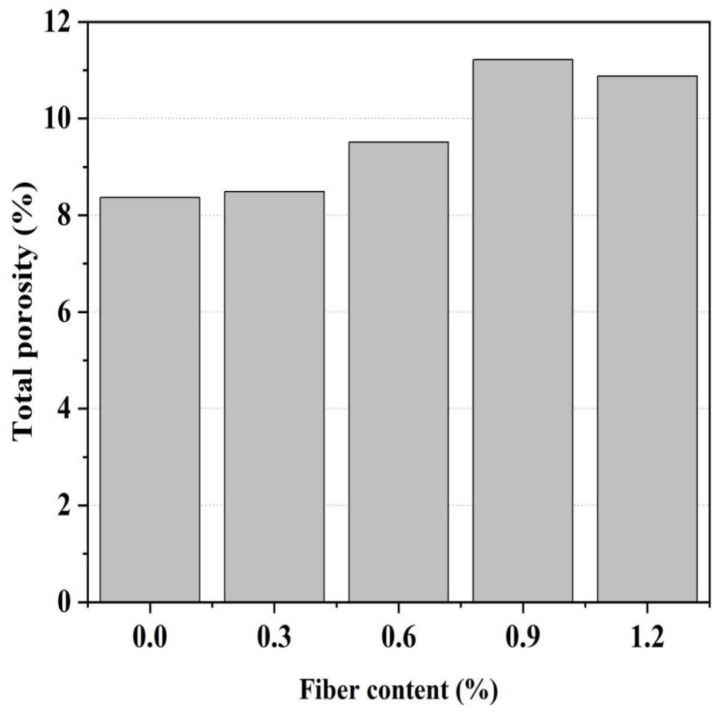
Porosity of MgO-SiO_2_-H_2_O cement with different fiber levels.

Figure 8 illustrates the pore structure characteristics of glass fiber-reinforced magnesium silicate hydrate cement-based composites, which indicate the principal pore size distribution in the MgO-SiO_2_-H_2_O system is in the range of 10–100 nm, and the inclusion of glass fibers does not significantly alter the pore size distribution in the cement mortar. The porosity of 28 d glass fiber-reinforced magnesium silicate hydrate cement composites with different fiber dosages (Figure 9) shows that the control group without any fiber content has the lowest porosity of 8.47%, while specimens with a 1.2% fiber content have the highest porosity of 10.88%. This result suggests that, as the fiber content increases, the porosity of the specimens slightly increases, which could be attributed to the thicker interfacial layer between the fiber and the cement matrix [43,44]. Despite the slight increase in porosity resulting from the addition of fibers, the glass fiber-reinforced magnesium silicate hydrate cement specimens demonstrate superior mechanical performance compared to conventional mortar, which is mainly due to the strong bonding force and lateral confinement between the fibers and the cement matrix [45]. Research has shown that the introduction of fibers to the cement matrix produces two primary effects: an increase in porosity and an enhancement of crack propagation resistance. If the increase in porosity outweighs the resistance to crack propagation, the strength of the composite material diminishes, and vice versa [46,47,48]. According to the results of this study, the addition of glass fibers enhances the strength of the composite material, indicating that the dominant effect is the resistance to crack propagation rather than the influence of porosity.

#### 3.2.3. SEM

**Figure 10:**
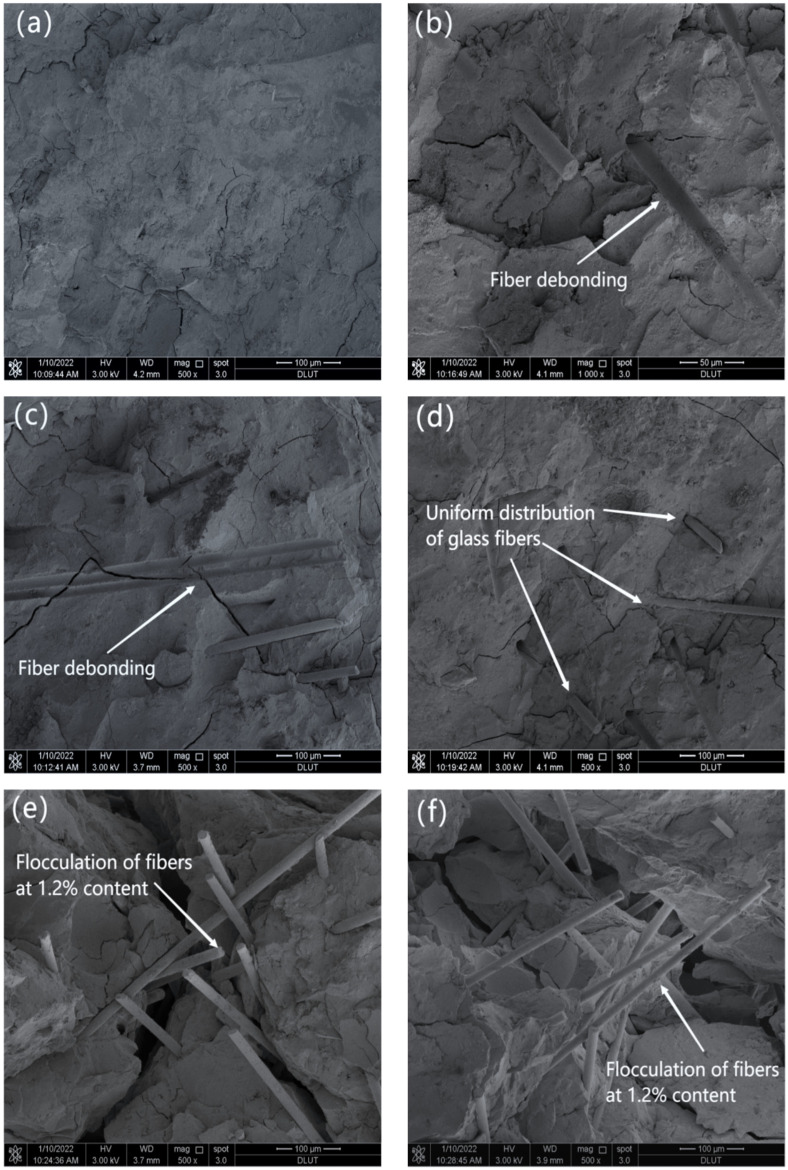
Micromorphology of specimens under different fiber content: (**a**) 0%, (**b**) 0.3%, (**c**) 0.6%, (**d**) 0.9%, (**e**,**f**) 1.2%.

In order to analyze the reinforcement mechanism of glass fibers in the MgO-SiO_2_-H_2_O cement, the surface microstructure of the specimens after flexural testing was observed using scanning electron microscopy (SEM). Figure 10a illustrates that the microstructure of the MgO-SiO_2_-H_2_O cement without glass fibers is more compact, which is consistent with the conclusion that the porosity of the specimens increases with the increase in fiber content. Figure 10b,c demonstrate the presence of a small amount of glass fibers, while Figure 10d shows that the glass fiber bundles are separated into individual filaments that are distributed randomly and uniformly within the cement matrix. The random distribution of glass fibers plays a critical role in preventing crack propagation, thereby providing further evidence for the improved mechanical properties of specimens with lower fiber content. When the fiber content reaches 1.2%, as shown in Figure 10e,f, the glass fibers exhibit agglomeration due to their uneven distribution, which explains the decrease in mechanical properties of the fiber-reinforced magnesium silicate cement mortar at high fiber content levels.

**Figure 11:**
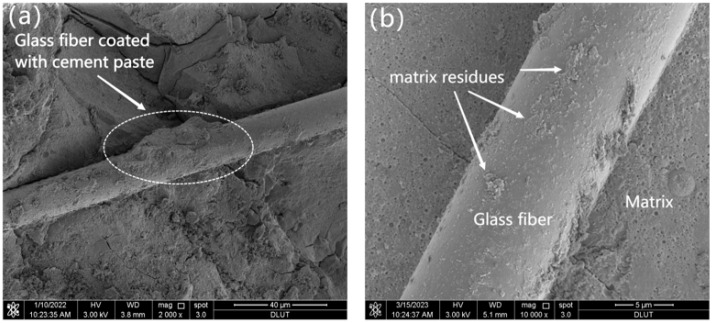
SEM images of M-S-H with 0.6% fiber content. (**a**) the magnification is 2000×, (**b**) the magnification is 10,000×.

From Figure 11a,b, it can be observed that the cement hydration products are bonded to the surface of the fibers, indicating a great chemical bond between the glass fibers and the MgO-SiO_2_-H_2_O cement. This bond enhances the friction resistance during fiber pullout, thereby improving the mechanical properties of the magnesium silicate cement [53]. The results suggest that the reinforcement effect of glass fibers on cement-based composites can be attributed to two main factors. One is that the glass fibers demonstrate good chemical compatibility with the cementitious materials. When glass fibers are added to cement-based composites, they tightly bond with the cement matrix, disrupting the original stress distribution and limiting crack formation. The other is that when cracks form, the glass fibers, as they traverse through the cracks, are capable of withstanding stress and act as bridges within the matrix, effectively preventing crack propagation.

### 3.3. The Shrinkage Properties

#### 3.3.1. Shrinkage Properties under Sealed Conditions

**Figure 12:**
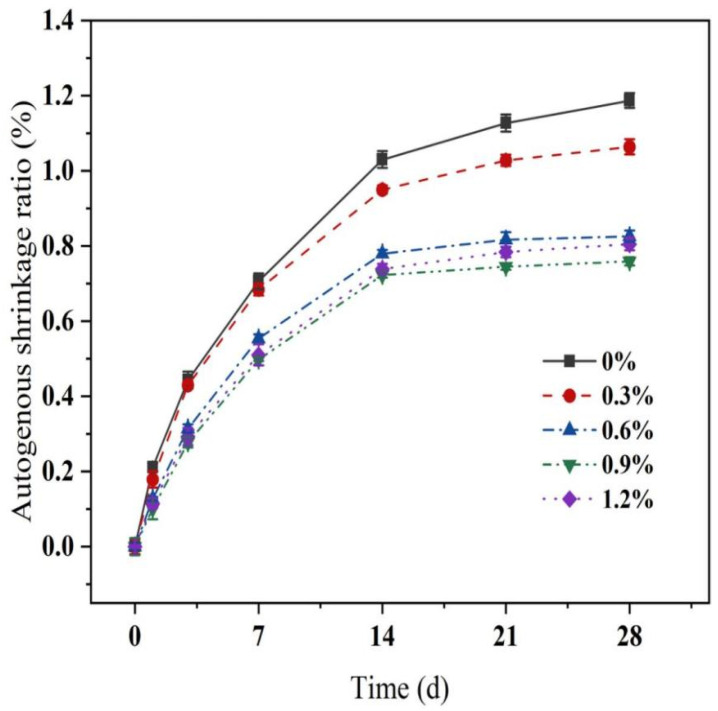
Effect of different glass fiber content on autogenous shrinkage rate of MSH mortar.

Figure 12 demonstrates the variation in the shrinkage of MgO-SiO_2_-H_2_O cement with different glass fiber contents under sealed conditions. The shrinkage rate under sealed conditions initially increases rapidly with the age of the curing period, and then the rate of shrinkage slows down after 28 days, but there is still a trend of continued shrinkage at 28 d. The addition of glass fibers effectively transmits capillary pressure, thereby reducing the shrinkage of the system. As a result, at the age of 28 d, specimens with fiber contents of 0.3%, 0.6%, 0.9%, and 1.2% exhibit shrinkage reductions of 11.5%, 30.4%, 36.1%, and 32.2%, respectively, compared to the control group. The self-desiccation shrinkage of specimens occurs under sealed conditions without any moisture exchange with the external environment, while maintaining a constant temperature. The primary cause of shrinkage is the high reactivity of MgO, which leads to rapid early hydration and increased water consumption, gradually reducing the internal relative humidity [62]. However, when glass fibers are added during the mixing process, they adsorb a small amount of free water on their surface and between individual fibers. As cement continues to hydrate, the pore structure gradually becomes finer, resulting in a decrease in internal humidity. At this point, the fibers release the absorbed water, slowing down the decline in internal relative humidity and restraining shrinkage under sealed conditions. In summary, experimental results indicate that the addition of fibers effectively reduces the shrinkage of magnesia–silica cement mortar under sealed conditions while enhancing its mechanical properties.

#### 3.3.2. Shrinkage Properties under Drying Conditions

**Figure 13:**
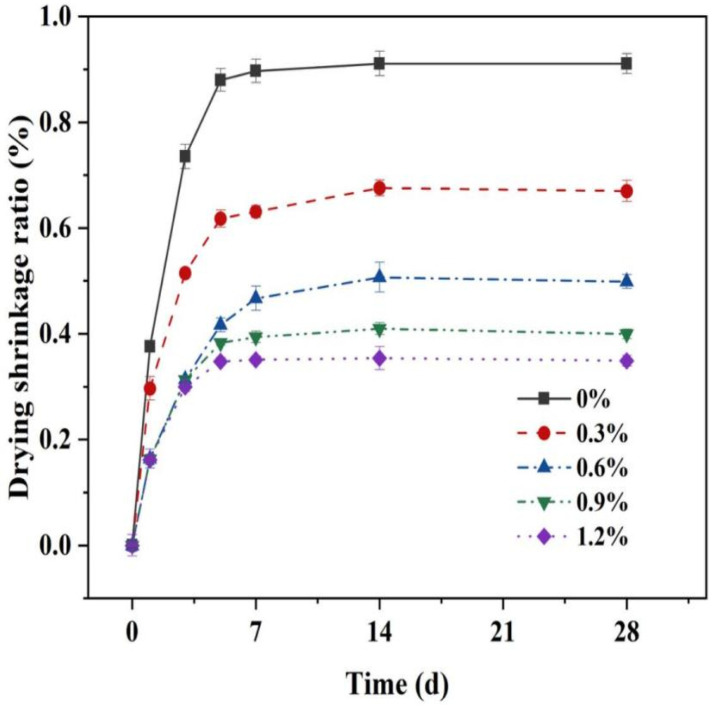
Effect of different glass fiber content on drying shrinkage of MSH mortar.

Based on the data shown in Figure 13, it is evident that the drying shrinkage curve of the test specimen exhibits two distinct stages. Initially, the drying shrinkage deformations develop rapidly before 7 days, at which point the drying shrinkage value accounts for more than 90% of the total shrinkage value; subsequently, the drying shrinkage stabilizes during the later stages. This is because the MgO-SiO_2_-H_2_O system has weaker water retention compared to ordinary Portland cement [54]. Under drying conditions, the internal relative humidity of the MgO-SiO_2_-H_2_O system decreases rapidly, leading to significant drying shrinkage. However, the introduction of glass fibers effectively reduces the drying shrinkage of the MgO-SiO_2_-H_2_O system. As the percentage of glass fibers increases (0.3%, 0.6%, 0.9%, and 1.2%), the drying shrinkage of the cement diminishes by 26.45%, 45.22%, 56.10%, and 61.70%, respectively.

The inhibitory effect of fibers on drying shrinkage can be explained by two main factors. Firstly, due to the high elastic modulus and random distribution within the mortar, the glass fibers efficiently transmit the capillary pressure generated by water loss and subsequent drying deformation within the cement matrix [63]. The incorporation of fibers in mortar creates a mechanical interlock between the fibers and the mortar matrix. The random distribution of fibers from multiple angles forms a supportive structure, which restrains the tensile stress generated by the internal shrinkage of the mortar and mitigates the overall shrinkage of the cementitious material [64]. Furthermore, the presence of fibers reduces the surface area available for water loss, thereby decelerating the rate of water dispersion [57]. Consequently, an appropriate quantity of glass fibers effectively reduces drying shrinkage while improving the dimensional stability of the system. Within the critical range, the fiber content increasingly constrains the cementitious material while also reducing the distance between glass fibers, thereby further enhancing the water loss area. Consequently, as the fiber content increases, the inhibitory impact of glass fibers on the drying shrinkage of the cementitious material becomes more pronounced.

Additionally, it was observed that the reduction effect on drying shrinkage becomes less significant as the fiber content increases. Specifically, when the fiber content rises from 0.6% to 1.2%, the drying shrinkage rate only decreases by 0.15%. When the glass fiber content reaches a critical threshold, an increase in fiber content leads to fiber agglomeration within the cementitious material, which in turn increases the internal pores of the cementitious material (as explained in Section 3.2.2) and reduces the inhibitory effect on drying shrinkage.

#### 3.3.3. Drying Shrinkage and Water Loss Ratio

**Figure 14:**
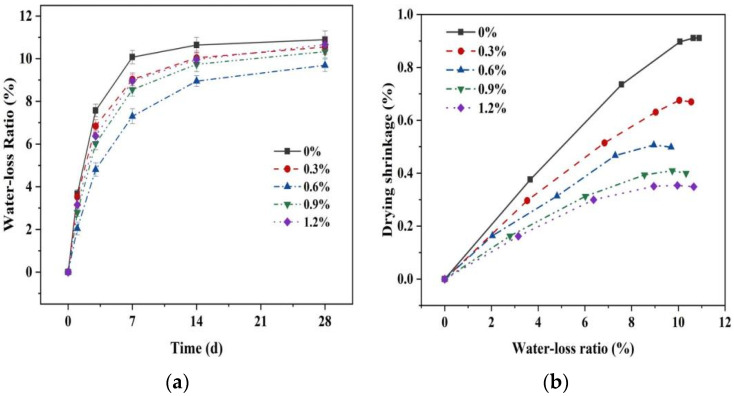
Water loss rate and time relationship of mortar with different fiber dosages: (**a**) the water loss ratio over time and (**b**) the water loss ratio and drying shrinkage.

Figure 14 demonstrates the water loss rate of the MgO-SiO_2_-H_2_O system with different glass fiber contents. The water loss rates increase as the drying shrinkage age progresses, particularly before 14 days, where the water loss rate variation is most noticeable, while after 14 days, the water loss rate remains stable. As depicted in Figure 14a, the addition of glass fibers can effectively reduce the water loss rate of magnesium silicate hydrate cement, which is consistent with the result that glass fiber addition can reduce the shrinkage of cement mortar. This can be explained by the distribution of glass fibers on the mortar surface, which decreases the contact area between the specimen surface and the external environment. Furthermore, the presence of glass fibers obstructs moisture channels within the mortar, limiting the pathways for water dispersion in the cement matrix and subsequently reducing the water loss rate. Figure 14b illustrates the relationship between the drying shrinkage rate and the water loss rate of MgO-SiO_2_-H_2_O cement mortar with varying glass fiber contents. The drying shrinkage rate increases as the water loss rate increases, and there is a linear correlation between the two parameters. This suggests that the growth trends and rates of both parameters are closely linked, indicating that the loss of internal moisture in the specimen under drying conditions is a significant factor contributing to the drying shrinkage of the mortar. As previous research has shown, the incorporation of glass fibers can reduce the rate of water loss during the cement hydration process by decreasing the surface area for water loss and minimizing the pathways for water loss, so that it effectively inhibits the drying shrinkage of the MgO-SiO_2_-H_2_O system.

### 3.4. Accelerated Aging Tests

The poor durability of glass fiber in ordinary Portland cement can be explained by the corrosion caused by the hydration of cement, which produces Ca(OH)_2_, leading to the deterioration of the fiber skeleton structure, reducing the enhancement and toughening effect, and ultimately compromising the structural strength and durability. However, the low alkalinity of MgO-SiO_2_-H_2_O cement mitigates the corrosion of the glass fibers. To evaluate the long-term durability of glass fiber-reinforced MgO-SiO_2_-H_2_O mortar, the specimens were cured under hydrothermal curing conditions at 20 °C, 50 °C, and 80 °C to assess changes in their performance under accelerated aging conditions. The analyses of XRD, TGA, and SEM were conducted to examine the hydration products and microstructure of specimens at different accelerated aging stages, allowing for the observation of glass fiber–cement matrix bonding and the assessment of glass fiber erosion characteristics. This study provides a theoretical foundation for expanding the application of glass fiber-reinforced magnesium silicate hydrate cement composites in various fields.

#### 3.4.1. Mechanical Properties

**Figure 15:**
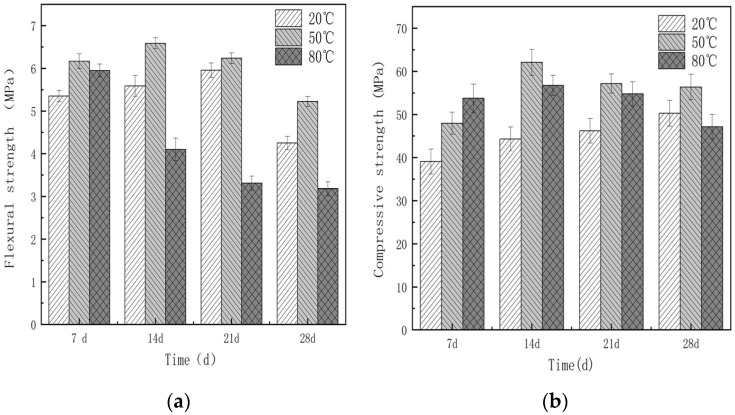
Mechanical properties of glass fiber-reinforced magnesium silicate hydrate cement at different curing temperatures: (**a**) flexural strength and (**b**) compressive strength.

Figure 15 illustrates the impact of the water curing temperature on the mechanical properties of glass fiber-reinforced magnesium silicate hydrate cement under different accelerated aging conditions. As depicted in Figure 15a, the flexural strength under room temperature conditions increases up to 21 d but decreases at 28 d. During the accelerated aging process, the flexural strength initially improves but undergoes a significant decline in later stages. Specifically, for specimens cured at 50 °C, the flexural strength surpasses that of specimens under room temperature conditions for the first 14 days and gradually decreases after 21 days, but still remains higher than that of room temperature-cured specimens. For specimens cured in an 80 °C water bath, the flexural strength increases at 7 d but experiences a remarkable decline after 14 days. Thus, it can be inferred that accelerated aging enhances the initial flexural strength of specimens at a relatively high temperature; however, with the progress of aging, the flexural strength exhibits a declining trend.

From Figure 15b, it can be observed that the specimens cured in water at 50 °C exhibit the fastest increase in early strength compared to those cured at 20 °C and 80 °C. The compressive strength at 14 d is 30.1% higher than that at 7 d. The strength of the samples cured in a water bath at 20 °C gradually increases with age; however, the compressive strength of the specimens cured at 50 °C and 80 °C gradually decreases at 14 d. Nevertheless, the compressive strength of the specimens cured at 50 °C remains slightly higher than that of the specimens cured at 20 °C. At 28 d, the compressive strengths of specimens cured at 20 °C, 50 °C, and 80 °C are 50.75 MPa, 54.17 MPa, and 49.55 MPa, respectively, which suggests that while increasing the curing temperature has a positive effect on the early strength of the specimens, it has an adverse effect on their compressive strength in the later stages.

The observed increase in early strength can be attributed to the appropriate curing temperature facilitating the reaction between Mg(OH)_2_ and SiO_2_, thereby accelerating the formation of M-S-H gel, and the increase in gel content contributes to the improvement in compressive strength. Research has also indicated that increasing the curing temperature enhances the polymerization degree of silicon–oxygen tetrahedra in M-S-H gel, promoting the transformation from Q^2^ to Q^3^ tetrahedra [65].

In summary, the mechanical properties of the specimens can maintain a basic level of stability in the early stages of accelerated aging under suitable temperatures. However, as the age increases, the mechanical properties start to decline. It should also be noted that excessively high temperatures, such as 80 °C, can have an adverse effect on the strength of the specimens. The reasons behind this decline in strength will be further analyzed in conjunction with the hydration products and microstructural characteristics.

#### 3.4.2. The pH Test

**Figure 16:**
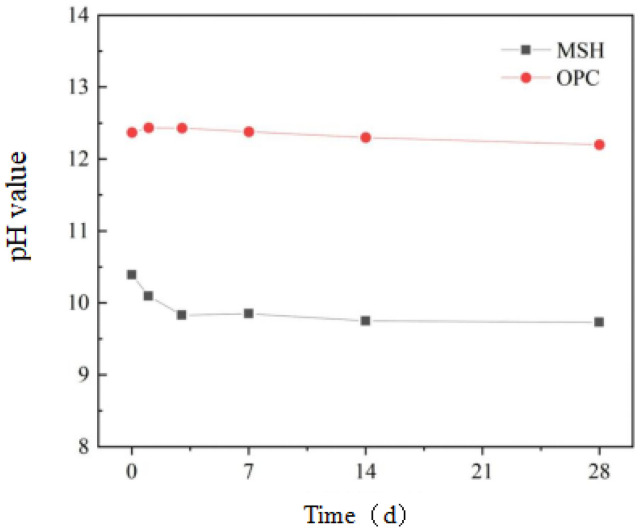
pH values of OPC and MSH cement at different aging ages.

The pH values during the hydration process of glass fiber-reinforced magnesium silicate cement under water-curing conditions at 50 °C were measured. As shown in Figure 16, the pH of OPC gradually increases with the curing age and stabilizes around 12.4 in the later stages of hydration. In contrast, the hydration process of magnesium silicate cement is primarily characterized by the consumption of SiO_2_ and the formation of M-S-H gel through the reaction with Mg(OH)_2_. After 14 days, hydration reaches a stable state, although there is a slow hydration rate between 14 and 28 days, resulting in a gradual decrease and stabilization of the pH around 9.83. Previous research [66] has confirmed that Ca(OH)_2_ produced during cement hydration reacts with SiO_2_ in glass fiber, forming hydrated calcium silicate and leading to the degradation of the silicon–oxygen framework in the fiber, thereby causing a reduction in fiber strength and reinforcement efficiency. Importantly, throughout the entire hydration period, the pH values of magnesium silicate cement are significantly lower than those of OPC, indicating a comparatively low level of corrosion of glass fiber by the magnesium silicate cement system.

#### 3.4.3. Characterization of Hydration Products

**Figure 17:**
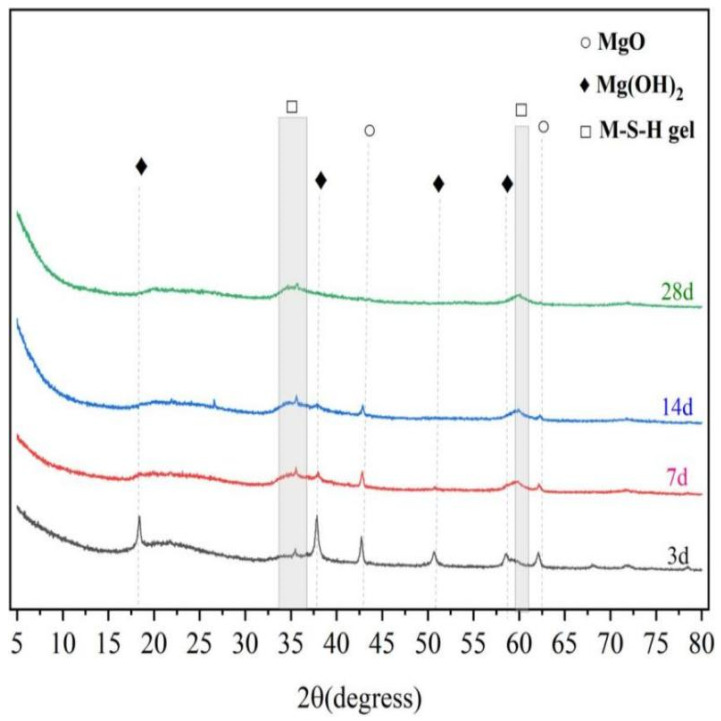
XRD diagram of M-S-H gel of different aging ages at 50 °C.

To investigate the hydration products of magnesium silicate hydrate cement under water-curing conditions at a temperature of 50 °C, X-ray diffraction (XRD) was used to characterize the hydration products at different stages of accelerated aging, as shown in Figure 17. The characteristic peaks of Mg(OH)_2_ are noticeable at 2θ = 18.6°, 38.0°, and 50.8°, and it is evident that the intensity of these peaks gradually diminishes with increasing curing age, which reveals that Mg(OH)_2_ is an intermediate product in the MgO-SiO_2_-H_2_O system and reacts with silicate ions during the later stages of the reaction, thereby forming M-S-H gel. The diffuse peaks of M-S-H gels are apparent at 2θ = 32–38° and 58–62° and become more pronounced as the curing age increases. Based on the XRD results, the accelerated aging experiment accelerates the hydration reaction, and throughout the long-term aging process, the types of hydration products of the MgO-SiO_2_-H_2_O system remain unchanged, with Mg(OH)_2_ and M-S-H gels as the main hydration products. Previous research [67] indicates that completely hydrated ordinary Portland cement (OPC) produces a substantial amount of Ca(OH)_2_ over its service life, thus maintaining a highly alkaline environment. The absence of Ca(OH)_2_ in the hydration products is the fundamental reason that the pH of the magnesium silicate hydrate cement system is lower than that of Portland cement, which also suggests that the MgO-SiO_2_-H_2_O system does not corrode glass fibers in the long term.

**Figure 18:**
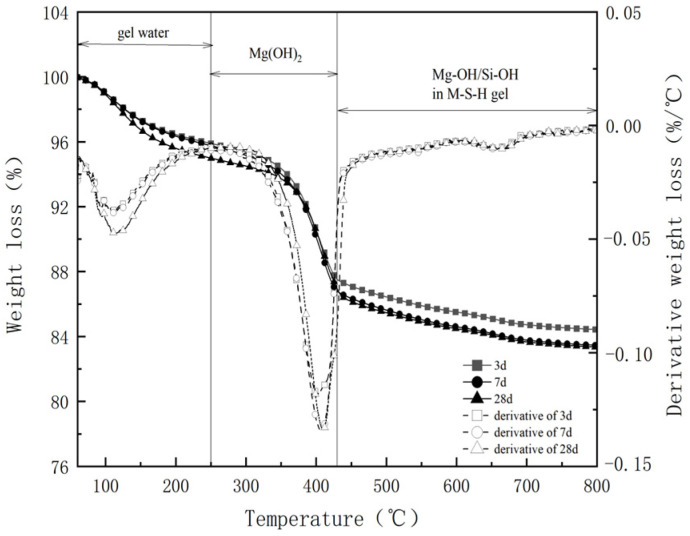
Thermogravimetric curves of different ages at 50 °C.

The hydration process of MgO-SiO_2_-H_2_O under 50 °C conditions was characterized using TGA testing, as depicted in Figure 18. This weight loss process can be divided into three stages within the MgO-SiO_2_-H_2_O system. The first stage, occurring between 50 °C and 250 °C, entails the removal of free water and physically adsorbed water from gel pores [68]. The second stage, between 250 °C and 430 °C, corresponds to the dehydroxylation process of Mg(OH)_2_ [69], which serves as an intermediate product in the hydration process of MgO-SiO_2_-H_2_O. During this stage, MgO undergoes initial hydration to form Mg(OH)_2_, followed by its reaction with SiO_2_ to form the M-S-H gel [70]. The third stage, between 430 °C and 800 °C, involves the removal of Mg-OH and Si-OH from the gel structure. Figure 18 indicates that changes in the hydration process under 50 °C conditions are not particularly noticeable with increasing aging. However, higher curing temperatures expedite the hydration process, and no new hydration products are observed throughout the entirety of the process. In summary, throughout the accelerated aging process, the M-S-H system consistently maintains a low alkalinity level, thereby ensuring the long-term presence of glass fibers in the cementitious materials.

#### 3.4.4. SEM

**Figure 19:**
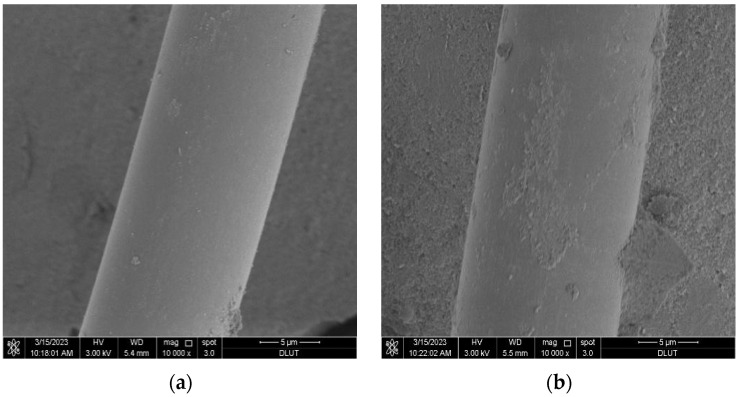

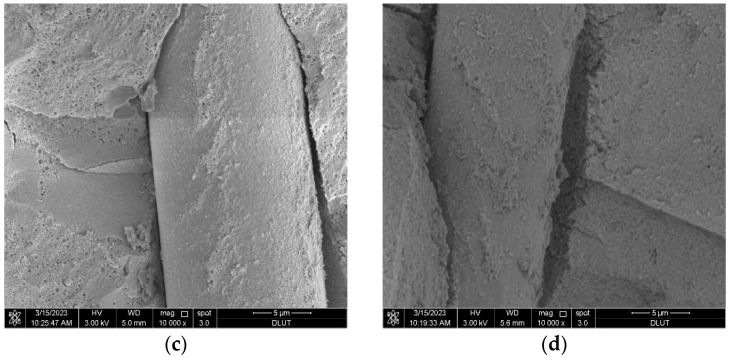
Morphology of glass fibers at different aging ages under the water-curing condition at 50 °C: (**a**) 7 d, (**b**) 14 d, (**c**) 21 d, (**d**) 28 d.

To investigate the potential of glass fibers to enhance the toughness of the MgO-SiO_2_-H_2_O system in the long term, SEM was used to examine the impact of accelerated aging on the interface composition between the MgO-SiO_2_-H_2_O system and glass fibers. Figure 17 presents the fiber morphology at different ages under water-curing conditions at a temperature of 50 °C. Figure 19a shows that the fibers remain mostly intact, with some cement debris attached to them. In contrast, Figure 19b reveals an increased amount of cement debris adhering to the fiber surface, but without any significant signs of degradation or erosion, which suggests that the MgO-SiO_2_-H_2_O system does not corrode the glass fibers and that the decrease in strength of the MgO-SiO_2_-H_2_O cement mortar is not a result of fiber corrosion. Figure 19d clearly illustrates that the interface structure between the glass fibers and the matrix becomes looser with increasing curing age, indicating a decrease in the bonding strength between the fibers and the cement. This leads to a reduced ability of the fibers to serve as effective bridge elements, which may explain the degradation of mechanical properties during the aging process.

## 4. Conclusions

In this study, glass fibers were used as a reinforcement material, and magnesium silicate hydrate cement was used as the matrix to prepare composite materials. The research sought to examine the effect of varying glass fiber content on the mechanical properties, shrinkage behavior, and aging resistance of the materials.

The main conclusions are as follows:

1. With an increase in the glass fiber content, the mechanical properties of magnesium silicate cement mortar, including flexural strength, compressive strength, bending toughness, and split tensile strength, initially increase and then decrease. Specifically, at a fiber content of 0.6%, the highest flexural strength and compressive strength were observed at 7.9 MPa and 42.5 MPa, respectively. At a fiber content of 0.9%, the flexural toughness steadily increases until reaching a maximum value of 2.238 N·m, which is 5.41 times greater than that of the control group. Within an appropriate range of fiber content, the addition of glass fibers effectively transmits and disperses the load, limiting crack development in the cementitious matrix and thus improving the mechanical properties of the cement mortar. This was confirmed by the analysis of pore structure and microstructure, which revealed that, while the addition of glass fibers increased the porosity of the cementitious matrix, the random distribution of fibers restricted crack development and enhanced mechanical properties. However, when the fiber content was too high, SEM results showed that the fibers tended to become tangled and agglomerated, leading to a decrease in mechanical performance.
2. Under sealed conditions, the incorporation of fibers effectively transmits capillary pressure and slows down the decrease in relative humidity within the cementitious matrix, thus inhibiting shrinkage. The inhibitory effect of glass fibers on autogenous shrinkage initially increases with increasing fiber content but eventually decreases. When the glass fiber content is 0.9%, the inclusion of glass fibers in the MgO-SiO_2_-H_2_O system demonstrates the most effective inhibition of autogenous shrinkage, as the reduction in autogenous shrinkage compared to the control group amounts to 36.1%. Similarly, under drying conditions, the addition of glass fibers significantly reduces the drying shrinkage of the cementitious matrix. Combining the results of water loss, it is evident that the drying shrinkage of magnesium silicate cement is positively correlated with the rate of water loss, and the inclusion of fibers reduces the rate of water loss in cement mortar, thereby suppressing drying shrinkage. When the glass fiber content is 0.3%, 0.6%, 0.9%, and 1.2%, respectively, the dry shrinkage reduction rates of MgO-SiO_2_-H_2_O cement mortar are reduced by 26.45%, 45.22%, 56.10%, and 61.70%. Moreover, the glass fiber inclusion enhances the confinement force on the cement, further suppressing drying shrinkage. As a result, the inhibitory effect of glass fibers on drying shrinkage improves with an increase in fiber content, while excessive fiber content diminishes the inhibitory effect on shrinkage due to fiber agglomeration.
3. Accelerated aging experiments were conducted by subjecting the specimens to water-curing at different temperatures, and the results showed that the glass fiber-reinforced magnesium silicate hydrate cement composite materials exhibited improved mechanical properties during the early stages of the aging process, but experienced a subsequent decline in properties. The analysis utilizing pH values, X-ray diffraction (XRD), and thermal gravimetric analysis (TGA) reveals that the hydration products of MgO-SiO_2_-H_2_O cement remain unchanged and maintain a consistently low pH value during the initial stages of accelerated aging, and the improvement in strength during the early stages was attributed to accelerated hydration and the promotion of M-S-H gel formation at higher curing temperatures. It can be seen from the SEM test results that a decrease in bonding strength between the fibers and the cement occurs, leading to a decline in mechanical properties.

## Figures and Tables

**Table 1:** Chemical composition and physical properties of MgO, silica fume, and glass fiber.

		MgO	Silica Fume	Glass Fiber
Constituent (%)	MgO	97.41	0.89	3.51
	SiO_2_	0.56	95.2	54.02
	Al_2_O_3_	0.03	1.04	13.35
	CaO	1.93	0.44	20.31
	Fe_2_O_3_	0.03	0.27	0.41
	Na_2_O	-	0.33	0.52
	K_2_O	-	1.23	0.37
Density (g/cm^3^)		3.16	2.18	2.68
Filament diameter (μm)				14
Tensile strength (MPa)				3300
Modulus of elasticity (GPa)				76

**Table 2:** Mixing ratio of glass fiber-reinforced magnesium silicate cementitious composites.

Group	MSH Binder (Mass Radio)			W/C	Volume Fraction (%)
	MgO	SF	Sand		
Ref.	1.0	1.5	2.5	0.55	0
GF-0.30					0.3
GF-0.60					0.6
GF-0.90					0.9

**Table 3:** Peak load, deflection, and flexural toughness of magnesium silicate cement mortar with different fiber content.

Fiber Content (%)	0	0.3	0.6	0.9	1.2
Load (kN)	1.344	1.714	1.921	2.809	2.455
Deflection (mm)	0.750	0.712	0.822	1.098	1.014
Integral Area (Flexure toughness, N·m)	0.413	0.628	1.296	2.238	1.654

## Data Availability

Since the experiment was completed with the support of Dalian University of Technology, the data used to support the results of this study are available from the responsible person and the author upon request.
